# Language and other artifacts: socio-cultural dynamics of niche construction

**DOI:** 10.3389/fpsyg.2015.01601

**Published:** 2015-10-20

**Authors:** Chris Sinha

**Affiliations:** School of Foreign Languages, Hunan UniversityChangsha, China

**Keywords:** biocultural niche construction, language, symbolic cognitive artifact, time concepts, human life course, social institutions

## Abstract

Niche construction theory is a relatively new approach in evolutionary biology that seeks to integrate an ecological dimension into the Darwinian theory of evolution by natural selection. It is regarded by many evolutionary biologists as providing a significant revision of the Neo-Darwinian modern synthesis that unified Darwin’s theory of natural and sexual selection with 20th century population genetics. Niche construction theory has been invoked as a processual mediator of social cognitive evolution and of the emergence and evolution of language. I argue that language itself can be considered as a biocultural niche and evolutionary artifact. I provide both a general analysis of the cognitive and semiotic status of artifacts, and a formal analysis of language as a social and semiotic institution, based upon a distinction between the fundamental semiotic relations of “counting as” and “standing for.” I explore the consequences for theories of language and language learning of viewing language as a biocultural niche. I suggest that not only do niches mediate organism-organism interactions, but also that organisms mediate niche-niche interactions in ways that affect evolutionary processes, with the evolution of human infancy and childhood as a key example. I argue that language as a social and semiotic system is not only grounded in embodied engagements with the material and social-interactional world, but also grounds a sub-class of artifacts of particular significance in the cultural history of human cognition. Symbolic cognitive artifacts materially and semiotically mediate human cognition, and are not merely informational repositories, but co-agentively constitutive of culturally and historically emergent cognitive domains. I provide examples of the constitutive cognitive role of symbolic cognitive artifacts drawn from my research with my colleagues on cultural and linguistic conceptualizations of time, and their cultural variability. I conclude by reflecting on the philosophical and social implications of understanding artifacts co-agentively.

## Introduction: Ecology, Evolution, And Niche Construction

The body is our general medium for having a world … Sometimes the meaning aimed at cannot be achieved by the body’s natural means; it must then build itself an instrument, and it projects thereby around itself a cultural world

(Merleau-Ponty, 1962, p. 146)

Niche construction theory is a relatively new approach in evolutionary biology (though with important but neglected precursors). It is regarded by many evolutionary biologists as providing a significant revision of the 20th century Neo-Darwinian “modern synthesis” that unified Mendelian genetics with Darwin’s theory of natural and sexual selection. Niche construction theory has in recent years been recruited to enhance explanatory accounts of cognitive, semiotic and language evolution (Deacon, 2003; Odling-Smee and Laland, 2009; Sinha, 2009, 2013, 2014; Iriki and Taoka, 2012; Stutz, 2014), as well as distributed cognition and abductive reasoning (Magnani and Bardone, 2008). I elaborate and extend these accounts here, to argue that niche construction theory lends support to a socio-ecological theory of language as simultaneously a biocultural niche and the foundational human social institution, and to offer an integrated account of biocultural evolutionary processes not only in the distant past of human ancestors, but also in historical time.

The most important features of niche construction theory for the purposes of this article are:

(1) It accomplishes a re-unification of Darwinian *evolutionary* theory with *ecological* theory, in which niche (or *Umwelt*, von Uexküll, 1957/1934), and organism-niche co-dependency, are key notions. In so doing, it contributes to the restoration to evolutionary theory of an integrative perspective largely neglected in the Neo-Darwinian synthesis. As Gontier and Serrelli (2014, unpublished thesis) put it “Adopting the Weismann barrier … caused a break with 19th century research on Epigenesis and Embryology, as well as early works in Ecology, General Systems Theory, and Cybernetics”. Alternative accounts, however, continued to be developed throughout the 20th century. Current developments in niche construction theory (Laland et al., 2000; Odling-Smee et al., 2003) can be seen as a continuation of the pioneering work of (Lewontin, 1970, 2000, 1983) and as complementary to other contemporary significant revisions of the Neo-Darwinian synthesis, such as evolution-development synthesis (“evo-devo”; Jablonka and Lamb, 2005; important precursors to these revisionist trends in can be found in Waddington (1953, 1977), Ho and Saunders (1984).
(2) It recasts the conceptualization of *agency* in evolution. Ecologists emphasize that species shape, as well as being shaped by, the niches that they occupy. In Neo-Darwinism “the agent of selection is the extra-organismic environment, including (a) the inanimate surround, (b) other species (a and b together being the basis of natural selection); together with (c) (subpopulations of) genes of the same species (the basis of sexual and kin selection) … This model, when appropriately formalized, can be extended by including cultural traits in the environment, that act as ‘amplifiers’ on the selection of genetic variation: this is known as the theory of gene-culture co-evolution (Lumsden and Wilson, 1981; Sinha, 2009, p. 292). Niche construction theory places an equivalent and complementary emphasis on the way in which organisms (and species) actively shape their environment (including the cultural environment), so that the dynamic of selection is driven by the behavior of the “selected” as well as the “selector.” Co-agency, as much as co-selection, is a crucial aspect of co-evolutionary processes.

This emphasis on the active organism is reminiscent of the psychobiological theories of Piaget (1979), who argued for the leading role of behavior in evolution; and Gibson (1979), in whose ecological psychology a key role is played by *affordances*: “properties of the ecological niche affording or supporting specific kinds of action made possible by the motor system and morphology of the animal. Such actions are both species-typical (though not necessarily species unique) and adaptive. Because affordances, Gibson (1979) maintained, are directly perceived, the phenomenal world of the animal is intrinsically meaningful, in that it potentiates the activation of perception-action circuits: objects present themselves as edible, climb-able, graspable and so forth” (Sinha, 2009, p. 294). Gibson did not, however, sufficiently stress the importance of the animal’s own behavior in the construction of affordances (Magnani and Bardone, 2008).

Such behaviors can significantly alter the environment to which the organism must adapt, initiating a process of positive feedback in which organism and environment are in a complementary, mutually shaping relationship. “A ‘path’ may … be an unintended consequence of locomotion from one place to another, but it is, nevertheless, a useful one … such shaping … can (however) introduce distal consequences—food shortage, erosion, pollution, competition with other species—which are outside the initial circuit of adaptation.” (Sinha, 1988, p. 136; see also Costall, 2004). Niche construction theory is built upon the recognition that the resulting niche can be construed as more than a contingent consequence of behavior. It is a *quasi-artifact*, to which the species is genetically, morphologically and behaviorally *adapted*, and which is integral to the evolutionary strategy of the species.

The term “quasi-artifact” signifies that, unlike the canonical case of human artifacts (analyzed below), such animal constructions need not be produced intentionally. Examples of animal quasi-artifacts are the nests of bower birds and the dams of beavers (Sinha, 2009, p. 294). The male bower bird builds and decorates an elaborate nest to attract females, using attractive objects such as flowers, shells and leaves. The bower forms an integral part of the male’s mating strategy, and sexual selection by the female is based upon the esthetic qualities of the bower, as well as upon the behavioral display of the male. Beavers construct dams, through coordinated and collaborative behavior, that both defend the colony against predators and enhance the availability of food. Beavers’ dams serve not only as a constructed, quasi-artifactual niche for beavers themselves, but also as a key factor in the maintenance of the wetland ecology enabling many other species to thrive. In suchlike cases, the behavioral repertoire of the species includes behaviors that are specifically adapted to the *making* of the quasi-artifactual niche, and these behaviors in turn support wider repertoires of behavioral strategies *exploiting* the niche. Quasi-artifactual niches are adaptive precisely *because of* the behaviors and strategies that they afford—nests are for nesting, and burrows are for burrowing.

A quasi-artifactual *material niche* can be regarded as an extension either of a behavioral repertoire (e.g., male mating display); or of the organism’s morphology (e.g., the bower bird’s bower is functionally equivalent, as a fitness indicator, to the tail of the peacock). Similarly, not only material constructions, such as nests or dams, but also species-specific *behavioral repertoires* (such as birdsong) can also be considered to be animal quasi-artifactual niches, inasmuch the song of the adults provides an environment within which singing behavior is epigenetically learned (Marler and Peters, 1982; Sinha, 2004). Such culturally transmitted, specialized behavioral repertoires are *biocultural* quasi-artifacts, functionally equivalent to, and constructively integrated with, material quasi-artifacts. There is no reason not to view human natural languages in the same way. This conclusion extends the proposal that hominid niche construction created *conditions favorable* for the emergence and evolution of language (Odling-Smee and Laland, 2009; Whiten and Erdal, 2012; Dediu and Levinson, 2013), by conceptualizing *language itself* as a symbolic biocultural niche/artifact (Sinha, 2009, 2013, 2014).

The ability to act in the biocultural niche of language (and the ability to learn how to act in it) involves not only the replication of phylogenetic adaptation to this niche, but the evolution and replication of an entire symbolically mediated *biocultural complex*. As Laland et al. (2000, p. 144) put it, niche construction “reestablishes the organism (or rather, classes of organism) as the central unit of human evolution, not as vehicle but as replicator. In fact, what is really replicated is a biocultural complex, with a composite array of information (acquired through multiple processes and stored at different levels) and inherited resources.” It should be clear that there can be no hard-and-fast distinction, from a biocultural perspective, between “niche” and “artifact”: a burrow, or a bower, are both artifacts and niches, and the biocultural complex includes the information, both in the genome of the organism and in the niche, that is required to learn how exploit it. This does not mean that the genetic information is a “copy of” or “blueprint for” for the artifact/niche; rather it is an adaptation to a constructive behavioral relationship between organism and niche. The same is, I suggest, true for language. The artifact/niche duality can be illustrated by a more recent example, namely the internet as a socio-semiotic ecology which is itself dependent upon the biocultural artifact/niche of language. The biocultural complex can be conceptualized as an evolving dynamic system comprising interlocking niches of different scale and granularity (see also Hutchins, 2013). I propose below, in regard to language and infancy, that the biocultural complex may include niche-niche interactions mediated by organismic evolution, as well organism-niche interactions mediated by organismic behavior.

Although other species than humans may display behaviors that can be regarded as both cultural and culturally transmitted (Whiten et al., 1999), human culture is distinguished by the predominant place occupied in the human biocultural complex by language—the foundation of what anthropologists call “symbolic culture,” and the semiotician Lotman (1990) called the *semiosphere*: the ensemble of linguistically based semiotic resources implicated in the establishment, maintenance and transformation of socio-cultural *habitus* (Bourdieu, 1977). Language may be the cornerstone of human culture, but it is also culturally situated, that is, it is dynamically embedded within the entire biocultural complex that includes other symbolic and non-symbolic artifacts. Treating language as a biocultural niche permits the unification, in a non-reductionist fashion, of the evolutionary dynamic of symbolic culture (the *semiosphere*) with that of material culture (the *technosphere*; Sinha, 2013). We should note, moreover, that the distinction between material culture and symbolic culture has been under increasing challenge in contemporary anthropology. As Boivin (2008, p. 190) has pointed out “Tools, technologies, and other aspects of the material world of humans and their predecessors have largely been seen as the outcome of evolutionary developments, and little attempt has been made to investigate their potential role as selection forces during the course of human evolution.” The same can be said of the biocultural niche of language, which is intricately interwoven with the other material and symbolic artifactual niche-structures that make up the human biocultural complex.

## Meaning And Materiality: Artifacts, Cognition And Semiosis

The materiality of meaning and meaningfulness of materiality is central to approaches in cognitive science emphasizing the importance of objects in extended cognitive embodiment (Sinha and Jensen de López, 2000); and in which cognition and communication are distributed over material-symbolic cognitive niches (e.g., Clark, 2006; Magnani, 2009; Sinha, 2014). All (human) artifacts are cognitive, inasmuch as they embody human intentionality (Bloom, 1996). Although the semiotic properties of artifacts have been comprehensively addressed in developmental psychology (e.g., Sinha, 1988, 2005; Rodríguez and Moro, 2002; Sinha and Rodríguez, 2008; Moro, 2011), the topic has received scant attention more widely in cognitive science. In general, artifacts have the following characteristics:

(1) Artifacts are made, not found. Although found objects may be used as tools, as with for example the sticks that chimpanzees use for “fishing” termites; or as constituents of artifacts, as with stones used by humans to construct dwellings and walls, artifacts (including artifactual tools) are produced by labor.
(2) Artifacts embody intentionality, conceptualization and imagination. An artifact is made according to a plan or design that involves the conceptual or imaginative representation by the maker of the finished article. It is this characteristic that distinguishes true artifacts from quasi-artifacts, and as far as we know the only species that produces true artifacts is *homo sapiens*, or as our species has also been aptly named, *homo faber.*
(3) Artifacts have canonical functions (Sinha, 1988) that are physically realized in the design features (or culturally produced affordances) of the artifact. The canonical function of an artifact is equivalent to the use value (Marx, 1976/1867) *for which it was designed*: *its socially standard function*. Non-artifactual (natural) objects or materials (such as wood or stone) may have use-values, but only artifacts have canonical functions. The canonical function of the artifact is *embodied in the artifact*. For example, the canonical function of a knife is to cut, the canonical function of a cup is to contain. The artifact can therefore be seen as embodying functional or relational concepts, such as CUTTING or CONTAINMENT, and these concepts are precisely those that are the objects of the design intentions of the maker.
(4) Artifacts *signify* their canonical function to a user who has the cognitive capacity to recognize the artifact as a token of a particular type (Sinha, 1988). The mode of signification that is intrinsic to the artifact is that of “counting as” (Searle, 1995; Tummolini and Castelfranchi, 2006). For example, a particular object (token) *counts as* a cup (type) if the perceiving subject recognizes the design features of the object (being a solid of a certain size and shape, having a cavity affording containment) as being those of a cup. This recognition of the signification relationship of *counting as* is a case of *perceiving as* – the subject *perceives* the object *as* a cup. If the object is not perceived as a token of a type having a canonical function, then it cannot be said to *count as* that type for the particular subject.
(5) To *count as* a type of artifact it is necessary for an object not only to afford the canonical function of the type (e.g., containment), but for this to be the *intentionally designed* canonical function of the token. For example, a half coconut shell can be used as a cup, but that does not make it a cup, unless it is *intended* to *count as* a cup, by virtue either of context or of baptismal naming.
(6) The *counting as* relationship, and the canonical function that defines the artifactual type, are *normative* and *cognitive.* They are aspects of normative and socially complex cognition. Canonical function depends upon, but is not reducible to, the physical properties of the object, since it is only by virtue of *some subset of* its physical characteristics (those that enable the object to be *perceived as* and *used as* a token of the artifactual type), and of their signifying value for the subject/agent, that the object counts as that artifact. We can thus compare artifacts with “institutional facts” (Searle, 1995), such as that a person is someone else’s sister-in-law, a social relationship that is also irreducible to the properties of the person’s physical body. In the next section, I analyze language as a *socio-semiotic institution*.

The characteristics listed above make it clear that artifacts are *cognitively and semiotically complex.* Artifacts (ranging from tools and vessels to notations and images) can be “read” (in the sense of “perceived as”), but (unless they are textual artifacts) they are *not* (contrary to influential postmodern theories) texts. The canonical functions that are served by artifacts are diverse, since they may be implicated in a wide range of cultural practices, both sacred and profane, including ritual, ornamentation, representation and narration, as well as technology.

Artifacts can support both non-representational practices (such as cutting and sewing) and representational practices (such as drawing and signposting). Although an artifact *embodies* and *signifies* its canonical function, it does not *represent* it (for a discussion of representation and signification, see Sinha, 1988, p. 44).The representational relationship can, following a long tradition in semiotics, be abbreviated as “X stands for Y in Context C”. This formulation expresses the relationship of “standing for” analogously with the abbreviation of the “counting as” relationship by Searle (1995) as “X counts as Y in Context C”. A full definition of representation is provided by Sinha (1988, p. 37): “To represent something –a scene, an event, an object, an interest—is to cause something else to stand for it, in such a way that both the relationship of ‘standing for,’ and that which is intended to be represented, can be recognized.”

To summarize: artifacts do not represent, or *stand for*, their canonical function, rather they *signify* it by *counting as a token of the type defined by that function*. Some artifacts, however, such as pictures, symbols and texts are also *representational*, embodying the *standing for* function *in addition to* the *counting as* function. The concatenation, in representation, of the *standing for* and the *counting as* relationships will be important for the analysis of language as a socio-semiotic institution in the following section.

Artifacts signify not only their canonical functions, but by extension the complexes of practices that they support (Sinha, 1988); and what is signified by the same object is not necessarily identical between different communities, contexts and universes of discourse. For example, Jensen de López (2002) found that young European children are biased in their actions directed to baskets by their perception and conceptualization of the object as a canonical container; while children from the indigenous Central American Zapotec culture do not display such a bias—a finding that was attributed to cultural differences in the habitual practices in which this class of objects are regularly used (Sinha and Jensen de López, 2000; Jensen de Lopez et al., 2005).

Artifacts, like words, may be polysemous in terms of what they signify and how they are perceived. For example, a smartphone may be perceived as just a token of the general type, but it may also be perceived as a token of a particular brand-name-defined type, and thus signify the social status and aspirational identity of its owner. The recruitment of objects-as-signs in interactive contexts is of great importance in ontogenetic cognitive development (Sinha, 2005). Given the status and complexity of artifacts as social-material signifiers, it is not surprising that the ontogenetic development of understanding of the canonical functions of artifactual objects, and their appropriation in practical and playful action, has a time course that is roughly correlated with the onset and consolidation of the acquisition of language (Sinha, 1988; Sinha and Rodríguez, 2008).

My prime concern here is with technological artifacts, that is tools or tool complexes whose canonical functions involve the amplification of the physical and/or mental powers of the agent: “Conceptualization of artifacts is a form of empowerment” (Tummolini and Castelfranchi, 2006, p. 311). Technologies may be classified in terms of the different kinds of powers that they amplify: motor (e.g., the hammer); perceptual (e.g., the telescope or telephone); or cognitive (e.g., the abacus). There is also, however, a further dimension in the typology of technological artifacts, namely the dimension of *augmentation* vs. *constitution* of the powers of the agent. Some technologies amplify the powers of the agent by *augmenting* already existing capacities and practices. For example, a bow and arrow augments the muscular power of the agent, enabling the arrow to be projected further and with a higher velocity than would be possible by throwing. Other technologies amplify the agent’s powers by *potentiating* and *constituting* entirely new practices. For example, a needle and thread potentiate sewing, a practice that would be impossible without the use of the technology, which can therefore be considered as *constitutive* of the practice.

Signs (including linguistic signs) and tools have frequently been compared. Bühler (1990/1934), influenced by the functionalism of Prague School linguistics, proposed the Organon Model of language (from the Greek Oργαανoν, meaning ‘instrument, tool, organ’. Vygotsky (1978/1930) also conceptualized signs as instruments, that not only enable communication between individuals, but also transform intra-individual cognition. Vygotsky (1978/1930) regarded the analogy as resting on the fact that both sign and tool support mediated activity; but he also distinguished between their *modes* of mediation: while tools, he argued, are “outer directed,” transforming the material world, signs are “inner directed,” transforming and governing mind, self and behavior (Vygotsky, 1978/1930, pp. 54–55). Vygotsky (1978/1930) emphasized the importance of semiotic mediation in transforming cognition and cognitive development, focusing on the internalization of conventional signs originating in contexts of discursive practice. He attributed great importance to the formative role of language in the emergence of “inner speech” and “verbal thought,” but his employment of the concept of semiotic mediation also encompassed the use of non-systematic signs, including objects-as-signifers. He paid little attention, however, to the role of culturally produced, linguistically grounded *symbolic cognitive artifacts* (see below).

Although I do not wish to advocate a technological determinist view of history, it is important to note that the socio-cultural consequences of practice-constituting technologies, and combinations of technologies, may be profound. Anderson (1991) discusses the emergence in the 16th–17th centuries of what he calls “print capitalism.” Mercantile capitalism based upon trade was not new, but the rapid dissemination of information made possible by print media, such as shipping lists and newspapers, paved the way for the emergence of the limited joint stock company, a new institutional form that transformed the world, ushering in the first era of economic globalization.

We might refer here, too, to the rather earlier invention of double-entry book-keeping as an accounting device permitting accurate recording and balancing of profits, losses, liabilities and assets. Double entry book-keeping is a good example of a *symbolic cognitive artifact*, the fundamental form of cognitive technologies (Norman, 1993). Double entry book-keeping is a technique for the ordering of symbolic (in this case numeric) information, in such a way that it permits the checking and auditing of accounts. It is not only desirable for individual traders, but it also provides necessary evidential support for the trust-based interpersonal relations involved in joint financial enterprises. Like other symbolic cognitive artifacts, it is a tool for thought (Waddington, 1977) that is transformative of both the individual mind and the shared, intersubjective mind.

Examples of symbolic cognitive artifacts are notational systems (including writing and numeric notations), dials, calendars and compasses. Symbolic and/or cognitive artifacts (Norman, 1993) have been plausibly proposed as key components of human cognitive evolution, in virtue of their status as external representations of cultural and symbolic practices (Donald, 1991). I will attempt to advance the argument further, by proposing that symbolic cognitive artifacts also have the status of agents of change in cultural-cognitive evolution, and are not mere repositories of prior changes in practices and cognitive structures and strategies. Cultural and cognitive schemas (Shore, 1996) organizing at least some conceptual domains may be considered, I shall argue, as *dependent upon*, and not merely *expressed by*, the employment of symbolic artifacts in cultural and cognitive practices.

To qualify as symbolic, an artifact must embody a *representational function* (Bühler, 1990/1934). Although all artifacts *signify*, inasmuch as their perceptible material form signifies their canonical function, only symbolic cognitive artifacts *represent* something outside themselves, through a sign function realized or embodied in the artifact^1^. All such sign functions are ultimately grounded in language, although they frequently (as in the case of compasses and maps) also incorporate iconic relations^2^. I define the class of *symbolic cognitive artifacts* as comprising those artifacts—which may either be entirely symbolic, such as number systems, or may embed or “anchor” symbolic information in material structures (Hutchins, 2005)—that *support symbolic and conceptual processes in abstract conceptual domains*. A key property of symbolic cognitive artifacts is thus that they are both *linguistically grounded* and *conventional*. Symbolic cognitive artifacts may be *motivated* by natural facts, and the human phenomenological experience of these facts, (e.g., the orbit of sun or moon; the number of fingers on a human hand), but they are not *determined* by them (witness, for example, the variety of arithmetical bases for number systems; Sinha et al., 2011, pp. 141–142).

**FIGURE 1:**
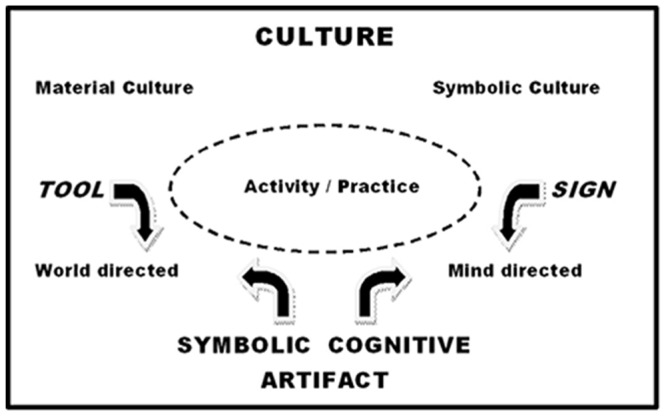
**The bi-directionality of mediated action employing symbolic cognitive artifacts.** Figure © Chris Sinha.

Symbolic cognitive artifacts are *both* world-transforming *and* mind-transforming (**Figure 1**). They are *tools* that afford and augment human interactions with the natural and social world; and they are simultaneously signs that *mediate* those interactions. As material anchors, and key nodes in the intersection of symbolic practice and material practice, symbolic cognitive artifacts also exemplify the co-construction and co-development of the interwoven semiosphere and technosphere. Symbolic cognitive artifacts, just as much as language, are constitutive parts of the human biocultural niche, and are of fundamental importance in human cultural-cognitive evolution. They afford symbolic systems and conceptual schemas that underpin the socio-cognitive practices (and the reproduction of these practices) constituting a segment of the life world (Schutz, 1966) of individual and group. The invention and use of symbolic cognitive artifacts is a crucial (and species-specific) aspect of the “ratchet effect” (Tomasello, 1999) in human cultural evolution^3^. The effects of such transformations on human cultural-cognitive-symbolic ecology may (as I argue below in relation to the calendar and the clock) be profound and (absent catastrophic social collapse) irreversible.

## Language As A Social-Semiotic Institution

Language, I have argued, is both a biocultural artifact/niche, and the semiotic foundation of symbolic cognitive artifacts. But that is not all. The symbolic cultures that human beings (and only human beings) have evolved are made possible by the social sharing of mind for which language is the principal vehicle (Zlatev et al., 2008). “Language is a social institution” (de Saussure, 1988/1916, p. 118) is one of the most oft-cited quotations attributed to Saussure (see also Stawarska, 2015). Language therefore has a dual ontology (Sinha, 2006), being both part of distinctive human biological species-being, and the foundational human social institution.

How can we employ the analysis I have offered above to clarify this nature of language as a social institution? My point of departure is the theoretical treatment of social facts and social institutions by two scholars whose work is separated by a century: the sociologist Durkheim (1982/1895) and the philosopher Searle (1995). I offer a semiotic interpretation of Searle’s theory, while characterizing the objectivity of language (and other social institutions) in Durkheimian terms; and derive from this discussion a formal definition of language that permits the differentiation of the traditional subsystems recognized in linguistic theory without recourse to a truth-conditional semantics^4^.

Durkheim (1982/1895) offered a theoretical and methodological clarification of social science and its object. This object he stipulated to be the domain of *social facts*, which he described as “a category of facts which present very special characteristics: they consist of manners of acting, thinking, and feeling external to the individual, which are invested with a coercive power by virtue of which they exercise control over him.” (Durkheim, 1982/1895, p. 30). Social facts, for Durkheim (1982/1895), are not merely aggregates of the individual cognitive representations of them by the subjects that are regulated, or “coerced,” by the social facts, since for each individual subject the social fact presents itself as a part of an out-there, already given *objective* reality. The objectivity of social facts consists, for Durkheim (1982/1895), in the fact they are independent of any single individual’s thoughts or will. As Jones (1986, p. 61) puts it, “it is precisely this property of resistance to the action of individual wills which characterizes social facts. The most basic rule of all sociological method, Durkheim (1982/1895) thus concluded, is *to treat social facts as things*.” Durkheim’s (1982/1895) treatment of social facts consists therefore in, first, an ontological proposition, that social facts are irreducible to biological or psychological facts (or structures or processes); coupled with, second, an epistemological and methodological proposition regarding their treatment: as *objects* of a particular kind, whose determinate nature consists in their “coercion” of conduct.

Durkheim (1982/1895) has often been criticized for the breadth and vagueness of his notion of “social fact.” A particularly problematic aspect of his theory is that, in counterposing “social facts” to “individual conscience” (or mind), he sometimes identified the former with “states of the collective mind.” Some social psychologists (e.g., Moscovici, 2000) have followed this direction in constructing a theory of “social representations,” but critics have claimed that Durkheim (1982/1895) sympathized with a view of society as a kind of super-organic “collective personality.” Whether Durkheim (1982/1895) believed in a “collective mind” or not, such a notion is not only scientifically dubious, it is unnecessary. I propose that a social fact can most simply be defined as something regulating an aspect of conduct which requires the *participation* (Goodwin and Goodwin, 2004) of more than one individual. This “something” may be a codified law, a norm, a canon of interpretation, an institution or a rule in the sense of Wittgenstein (1961). Social facts, for mature human beings, are objects of *common knowledge*; language is a prime example of this (Lewis, 1969; Itkonen, 1983; Clark, 1996). However, the social fact itself is not the sum, average or common denominator of all the individual beliefs of participants (since it is, indeed, the *object* of these beliefs). Social facts, in this sense, are in some way prior to individual cognitions about them. Yet it cannot be claimed that social facts are *independent* of cognitions, since their normative status is dependent upon agreement in cognition. We shall return to this paradoxical problem in discussing Searle’s theory of social facts.

Given that non-human species also display social behaviors, should we regard social facts as being uniquely human? Ethologists have pointed to the evolutionary roots of norms, rules and conventions in the ritualized displays that many species exhibit in, for example, mating and agonistic displays. Ritualization, in turn, can be regarded as falling under the definition of a biocultural niche as discussed above. If so, we could argue that social facts are nothing other than biocultural niches regulating and sustaining, supporting, and constraining, the participatory behavior of more than one individual. This definition is entirely consonant with Durkheim’s (1982/1895) view that social facts “consist of manners of acting, thinking, and feeling external to the individual, which are invested with a coercive power by virtue of which they exercise control over him.”

Under this interpretation, social facts would be seen as no more unique to humans than culture. Yet there is clearly something unique about human social facts. This uniqueness consists, surely, in the way in which social facts are cognitively constructed as *objects* of intersubjective common knowledge, common commitment, and common emotional investment, so that they can be *known* in the way in which the rules of football, the laws of the land, or a family history may be known. To bring some order into the definitions and terminology employed here, I will use below the term “social institution” to refer to systems of social facts. I will further stipulate that the concept of “social institution” pertains to social knowledge systems that are of a fully normative nature; that is, those which not only regulate behavior, but are known to do so, and knowledge of which (whether explicit or tacit) is essential to their regulative status. Social institutions, on this definition, can only be *constructed* by human beings with a certain level of cognitive development, although they may be participated in by animals which lack this cognitive status (e.g., prelinguistic infants in language practices, racehorses in horse races etc*). Social institutions, then, constitute an emergent ontological level*, within the wider category of biocultural niches, and one which is uniquely human.

Searle (1995) situates knowledge and belief at the heart of his account of social (or institutional) facts: “There are things that exist only because we believe them to exist. I am thinking of things like money, property, government, and marriages … (such) Institutional facts are so called because they depend upon human institutions for their existence” (Searle, 1995, pp. 1–2). Searle’s (1995) account of social or institutional facts (such as money) is that they depend upon collective agreement and knowledge that, under determinate rules, something *counts as* an instance of a social object. The general form of such rules is:

(1) “X counts as Y in context C” (Searle, 1995, p. 28).

Although he never uses the term, Searle’s (1995) definition is a semiotic one, in that the “counting as” relationship is one of *meaning* or *signification*. The twenty dollar bill, for example, signifies a certain monetary value or equivalence. However, the relationship between the bill and its monetary value is not a fully fledged *sign relationship*. The bill does not *represent* or *stand for* twenty dollars: it simply *is* twenty dollars, it is self-identical to its monetary exchange value. To clarify this difference, we can point out that the numeral 20 printed on the bill *stands for* (represents) the number twenty, but the bill itself does not represent, for example, 20 (tokens of) one dollar bills, but rather is *equivalent* to them in value. *Representation*, in contrast, involves more than equivalence and categorization. Sinha (1988) defines the pragmatic and semiotic *conditions on representation* as follows: “To represent something … is to cause something else to stand for it, in such a way that *both* the relationship of ‘standing for,’ *and* that which is intended to be represented, can be recognized” (37). Intrinsic to the conditions on representation is a *duality of cognition*, paralleling the duality of sign structure (the conventional unity of signifying substance and its signification). Two cognitions are necessitated: the recognition of the *sign relationship*, and the recognition of *what is signified*. The “counting as” relationship, by contrast, has no such duality: to know that something counts as a particular object, however, abstract or complex that object may be, it is necessary only to recognize it as a *token* of that category of objects.

What is necessary to grasp the “counting as” relationship is knowledge of the rules and norms that constitute the category (for example money, or a language). In one fundamental (if limited) sense, then, knowledge of a language is knowledge of what *counts as* a token of the language, and in order to know this, the knowing subject must necessarily know (in some way and to some degree) the rules of the language. It is this level of knowledge that is considered to be primary in generativist and other formalist theories of language, whose goal is to elucidate the rules that constitute (or generate) the full range of tokens for which it is the case that:

(2) X counts as (a sentence) S in L (a language)

This definition does not, however, touch on the *representational* function of language (Sinha, 1988; Bühler, 1990/1934). That is, the capacity of the *linguistic symbol in use* to represent things (situations, events, actions, objects) *outside* the formal context of L: that is, the world outside language. Knowledge of (2), in itself, does not enable the knower to *use* the language representationally, any more than knowledge that a piece of paper is a twenty dollar bill in itself enables the knower to use the bill in financial transactions. Knowledge of (2) is (akin to) knowledge of language in the narrow sense (Hauser et al., 2002): it is a necessary but not sufficient condition of being a language user, since it encompasses neither semantics not pragmatics.

The knowledge constituting the *semantic* domain is governed, not by the “counting as” relationship and its conditions, but by the “standing for” relationship” and its conditions. This “standing for” relationship can be notated, in a way parallel with Searle’s (1995) notation of the “counting as” relationship, as follows:

(3) S (a sign) stands for M (a message) in context C

However, the duality inherent in the conditions on representation (above) requires that this preliminary notation be expanded, to include knowledge on the part of the subject that S *counts as* a sign, or, more accurately, that a particular object counts as a signifier. This expansion yields:

(4) [X counts as S and S stands for M] in C

Where X is a token of the class of signifiers in C

(4) is sufficiently general to cover all cases of sign use, including highly idiosyncratic and context bound cases, such as non-conventional gestures. It is not restricted to language. We can now undertake a further expansion to specify cases in which a given sign is part of a *conventional sign system*, shared by a particular community of users:

(5) [X counts as S and S stands for M in C_s_] for C_u_

Where:

C_s_ = conventional sign system

C_u_ = community of users

In the specific case of language, we can reduce the notion of a *sign system shared by a community of users* to the simple term L, language, thus:

(6) L = C_s_ for C_u_

This is equivalent to the statement that a language L is a conventional system of signs used by a community of users. Now any grammatical and conventionally meaningful instance of language use X can be expressed as follows:

(7) [X counts as S and S stands for M] in L

Note that, consistently with the approach of Cognitive Grammar (Langacker, 1987), S (the signifier) is an expression at any level, sub-lexical, lexical or constructional; grammatical assemblies of signs are also signs. The definition offered in (7) can thus be considered to be the notational reduction of the broader theoretical approach to language taken by cognitive and functional semantically based theories, and indeed by all linguistic theories that include representational meaning in the linguistic theory. It is clearly a more inclusive definition than the formal-sentential definition (2), reproduced here:

(2) X counts as (a sentence) S in L

Definition (7) is also, quite simply, more psychologically complete than (2): what we usually mean by “knowing a language” is the knowledge of *both* what counts as a token of the language, *and* what it means (stands for) when *standardly used*. In concluding this section, I will attempt to elucidate further just what is, and is not, necessary for such knowledge. Before doing so, I pursue this formal-notational exercise further by exploring how the conjoint definitions of “counting as” and “standing for” can be employed to define the sub-systems of language as traditionally employed in linguistic theory.

**Grammar** (in the wide, cognitive grammar sense, including lexical form and phonology) can be defined as:

(8) X counts as S in L

X is an instance of S, and S is a grammatical expression in L. The distinction between X and S is the distinction between, for example, phonetics and phonology.

Presupposing (8), **semantics** can be defined as:

(9) S stands for M in L

This is the relation between, for example, word form and lexical entry or concept; or, more generally, between linguistic expression and linguist conceptualization. What, however, of pragmatics? The Gricean account of the distinction between semantics and pragmatics (Grice, 1989) is summarized by Kempson (1988, p. 139) as follows: “Semantics provides a complete account of sentence meaning for the language, (by) recursively specifying the truth conditions of the sentences of the language … Pragmatics provides an account of how sentences are used in utterances to convey information in context.” Cognitive linguists reject the truth-conditional account of linguistic meaning, and with it the distinction between pragmatics and semantics, in favor of an account based upon convention and entrenched usage. As Langacker (2004, p. 70) puts it, there is “no a priori reason to accept the reality of the semantics/pragmatics dichotomy.” He argues that this is because there are “gradation(s) of centrality in the specifications constituting our encyclopedic knowledge of an entity.” In other words, he argues that one cannot reliably distinguish “core meaning” from encyclopedic meaning, since all usage is to a greater or lesser extent encyclopedic.

However, a distinction may be real even if it is granted that it is vague, or even undecidable, since along with undecidable cases there may be clear-cut cases. Itkonen (1978, p. 108) makes this point clearly and forcefully: “All distinctions which either involve social life... or obtain in it are relative … However, … even if each of the distinctions concerned forms a continuum, the end points of such a continuum are ABSOLUTELY DIFFERENT (in the relevant respect).” The definition of semantics that I have offered in (9) above does not depend upon a truth conditional account of meaning: it is based upon *meaning in conventional usage*. How can we extend and modify this account to incorporate non-conventional, contextually determined variations in meaning, the traditional domain of pragmatics?

Presupposing (9), **pragmatics** can be defined as:

(10) S counts as A_s_ for Participants_(2__…n)_ in C_d_

Where:

A_s_ = Speech act (including reference)

C_d_ = Discourse context

Under this description, pragmatics is the closest of the linguistic subsystems to a pure instantiation of the “counting as” relationship. This accords with the intuition that pragmatics is not “systematic” in quite the same way as grammar and semantics; that speech acts are specifically linguistic instances of more general communicative acts (such as “threats” and “invitations” in both human and non-human species); and that their interpretation is strongly dependent on gesture, prosody, posture, physical, and linguistic context.

Having employed the notational formalism to distinguish the subsystems of language one from another, we can now re-assemble them to analyze the structure of particular utterances in their context.

(11) [X counts as S and S stands for M] in L and S counts as A_s_ for Participants _(2__…n)_ in C_d_

Such a re-assembly does not yet account for the *interaction* between semantics, pragmatics, extra-linguistic context and shared world knowledge in actual utterances. For example, if the utterance is “You really did well this time!”, and it is clear from the context that the speech act is one of ironic praise, the contextual meaning is “You did very badly.” Or, if the utterance is “The road meanders up the hill,” the contextual meaning is that the road has a winding path, not that the road is itself in motion (Talmy, 1996). How can we capture such facts of language?

It seems impossible to do so without appealing to psychological processes such as inference, default and prototypic reasoning, subjectivization and perspectivization (Langacker, 1987). If we wish to formalize this, it would look something like this:

(12) [X counts as S and S stands for M] in L and S counts as A_s_ in C_d_

=>S counts as (having) M_c_ for H in C_d_

Where:

M_c_ = Contextual meaning

H = Hearer

C_d_ = Discourse context

This brings us back, in an intriguing hermeneutic circle, to Searle’s (1995) original definition of a social fact, and emphasizes the truism that, in the end, all meaning is contextual and situated. This does not, however, mean the same as saying that there are no institutionalized, relatively stable, relatively autonomous and systematic linguistic facts; indeed, it is precisely this very relative stability and autonomy which constitutes the *objectivity* of social facts emphasized by Durkheim (1982/1895).

This objectivity is not to be confused with the *objectivism* of formal, truth conditional semantics. Amongst the advantages of the simple notational definitions developed here are:

• The account of semantic meaning is underdetermined by this formulation. The semantic theory need not be truth-functional, but *is* (necessarily) conventional and normative (as indeed are all the subsystems).
• Semantics is distinguished from pragmatics without necessitating a truth functional semantics.
• Contextual dependence characterizes all subsystems, as well as the interactions between them, but does not erase the distinctions between them.
• Language has its own proper structure which necessitates, but is irreducible to, the intentionality of its users. Language, like all social institutions, is an *objectification of intersubjectivity*, with an emergent structure relatively autonomous from the intentional states (such as mutual knowledge of the language) which are possessed by its users. It is in this fact, and this fact alone, that the objectivity of language inheres.

What implications does this analysis hold for theories of the human language capacity and language acquisition? Although there can be no scientific objection to the study of language as a purely formal system, insistence on the disciplinary autonomy and full explanatory adequacy of formal theories leads to a distorted picture of the human language capacity, and to unnecessarily constrained theories of language acquisition. If “knowledge of language” is restricted to knowledge of what counts as a grammatical sentence, not only is language itself as a semiotic system truncated and reduced, but the process of its acquisition is rendered incomprehensible. It is to fill this conceptual vacuum that innate knowledge of Universal Grammar has been invoked (Chomsky, 2000).

Furthermore, the identification of *language* with *knowledge of* language is a fundamental error, involving a conflation of ontology and epistemology. Language as a social institution is an (inter)-objectification of intersubjectivity. It cannot be identified with inter-individually variable individual knowledge either of the “how” of participation in language practices, or of the “what” of the language system that normatively regulates such participation. This is not a new point. It was emphatically stated by McNeill (1979, p. 293) who wrote: “Grammars refer to real structures, though not to psychologically real structures in the processing sense … a grammar is a description of our *knowledge of a social institution*—the language—and because of this basis in social or institutional reality, rather than in cognitive functioning, grammars and psychological processes have no more than (a) loose relationship … The role of grammar during speech programming is analogous to the role of other social institutions in individual behavior. This role is to define and evaluate the behavior of individuals. It is not to cause the behavior.”

I would wish to qualify this satisfyingly prescient quote by emphasizing that (a) “our knowledge of a social institution” should be understood as “analyst’s knowledge,” rather than “speaker’s knowledge,” (b) “define and evaluate” should be understood, in the influential terms introduced by Searle (1969), as “constituting *and* regulating”; and (c) the irreducibility of the rules of language (or any other system of norms) to individual cognition of these rules does not mean that individual cognition plays no part at all in their emergence, development, and evolution (Boella and van der Torre, 2004).

Language is both a biocultural niche, and a social institution normatively regulating linguistic practice, and it is the practical ability to adhere to its conventions that is acquired by the language learner. From this perspective, “knowledge of language” is both richer, in one sense, and poorer, in another, than that to which we have become accustomed from generative linguistics. It is richer because it incorporates meaning and context, the fundamental pillars supporting both language acquisition and language use. It is poorer because there is no longer a compelling reason to attribute a knowledge equivalent to the results of formal analysis to the learners and users of language. Simply stated, in the biocultural approach, *there is no mental grammar* isomorphic with autonomous grammar. The learner need not internalize a formal description of the structure in order to acquire the ability to *act* in it as a participant. Language is not an “input” to a processor or device, but a structured niche affording complex and semiotically mediated communication, cognition and participation. The capacity to learn language, although it is almost certainly supported by genetic adaptations to the biocultural niche of language, is not innate, but epigenetically developed (Sinha, 1988, 2004, 2014).

## Epigenesis, Enchrony, And The Extended Human Life Course

Epigenesis and epigenetics are terms referring to inheritance processes and mechanisms, at different levels ranging from the molecular to the organismic, that are controlled or modulated by factors other than those inscribed in the genome (Jablonka and Lamb, 2005). Epigenetic developmental processes in ontogenetic behavioral development are those in which the developmental trajectory and final form of the developing behavior are a consequence as much of the environmental information as of the genetically encoded information. A genetically specified initial behavioral repertoire is subsequently *elaborated* through experience of a relevant environment, yielding an envelope of potential trajectories and outcomes

(Sinha, 2013, pp. 264–265).

The process of elaboration is directional, and once it has taken place the initial plasticity of the embryonic, or unelaborated, repertoire is largely (though not necessarily wholly) lost. In other words, epigenesis involves a developmental transition from relative organismic plasticity and informational openness, to relative rigidity and informational closure. Augmented epigenesis can be hypothesized to be advantageous for organisms in which *phenogenotypic organism-niche couplings* are both frequent and variable, which is an appropriate general description of the human biocultural organism. Regulatory genes augmenting epigenetic openness can therefore be expected to have been selected for in the human genome, permitting further adaptive selection for domain-specific learning in the biocultural complex, in particular for language. Although I do not reject the possibility that the epigenetic processes selected in the evolution of the human biocultural complex include a predisposition for learning syntax, this does not necessarily imply that any such predisposition is or was “dedicated” from the start exclusively to language, and it certainly does not imply anything resembling an innate Universal Grammar. In an epigenetic perspective, any adaptive developmental predisposition for learning language is unlikely either to involve direct coding of, or to be dedicated exclusively to, linguistic structure (Mueller, 1996). Rather, we may hypothesize that epigenetically governed adaptations initially evolved in response to prelinguistic socio-communicative processes.

Infancy, childhood, and adolescence are not merely biological stages of organismic development. They are also developmental stages of the human-specific biocultural niche supporting mutual, intersubjective, emotional, communicative, and cognitive engagement (Trevarthen, 1998). In the course of human development, *the proximal environment of the organism develops along with the maturational processes underlying the individual’s development*. This developmental, and developing, niche includes as its most important component other human beings, including the caregiving adults and older children who participate in the co-construction of its material, communicative and symbolic properties. This co-construction process involves (a) the spatio-temporal organization of settings involving joint actions contributing to the reproduction of socio-cultural habitus (Bourdieu, 1977); and (b) the scaffolding and shaping of interactions in the niche on the part of the caregiver (Wood et al., 1976; Ninio and Bruner, 1978), in response to both the actions and the competences of the developing child. These fundamental processes in niche construction and niche development can be thought of as involving the cultural and situational structuring of affordances for learning, and are the socio-developmental mechanisms supporting inter-generational cultural transmission in all human cultures.

Social learning of complex skill repertoires, including language, is optimized by the extended childhood of our species. As Arbib (2012, p. 166) observes, “the prolonged period of infant dependency … combines with the willingness of adults to act as caregivers and the consequent development of social structures to provide the conditions for complex social learning.” The biology of ontogenetic life history is assessed in comparative research by physiological and anatomical markers. There is a general consensus that the human extended life history did not appear until the emergence of later members of the genus Homo (Dean, 2007; Robson and Wood, 2008; Lee, 2012; Schwartz, 2012). Recent research suggests, moreover, that the early stages of modern human (*sapiens*) life are uniquely extended, even in comparison with Neanderthals (Smith et al., 2007a,b). The earliest evidence of an extended human childhood with a life history profile similar to that of contemporary children is from modern human child remains from 160,000 years ago (Smith et al., 2007a). Smith (2013, p. 203) concludes that “the characteristically prolonged development of living humans fully evolved after (modern humans and Neanderthals) diverged,” signaling the “advent of corresponding social, biological, and cultural changes necessary to support highly dependent children with prolonged opportunities for social learning in early childhood” (Smith et al., 2007a, p. 6132).

Evolutionary and developmental processes take place on different time scales or *durées*, of phylogenesis, sociogenesis (or sociocultural evolution), ontogenesis, and microgenesis (Valsiner and van der Veer, 2000). Although, as I stressed above, the niche develops along with the developing human being, the time scale that principally characterizes niche construction and niche development processes is not that of ontogenesis, but that of microgenesis. Microgenesis is a term coined by Werner (1957) to denote developmental advance through re-organization, occurring in the time scale of actual engagement with a problem to be solved. Microgenesis as individual or collaborative situated learning and development also implicates the time scale of actual communicative interaction, labeled *enchrony* by Enfield (2011, 2013), in distinction to the traditional structural linguistic distinction between diachrony and synchrony. “An enchronic perspective on human communication focuses on sequences of interlocking or interdependent communicative moves that are taken to be co-relevant, and causally conditionally related. Enchrony is a level of temporal-causal grain (typically, ‘conversational time’) that an analyst of communication can adopt, as distinct from other possible perspectives, fitted to other purposes, that focus on other temporal scales and other kinds of causal-conditional process; these include phylogenetic, diachronic, ontogenetic, epigenetic, and synchronic perspectives.” (Enfield, 2011, p. 287; see also Steffensen and Pedersen, 2013).

Enchrony, in Enfield’s definition, is prototypically conversational temporality. Enchronic interactional coordination is manifested, however, long before the emergence in development of language, and is a fundamental property of infant as well as adult communicative meaning-making, probably implicated in both musical and narrative production (Trevarthen, 2008). It can also be argued, however, that the notion of enchrony can equally be applied to the planning, sequencing, and timing of component actions whose combination makes up complex praxic actions (Arbib, 2012), including cooperative praxis. If this is correct, enchrony is, in general, the *durée* that most appropriately characterizes human skilled action and interaction, and that is in at least some circumstances co-temporaneous with microgenesis, the appropriation and mastery of new actions and new communicative resources.

**FIGURE 2:**
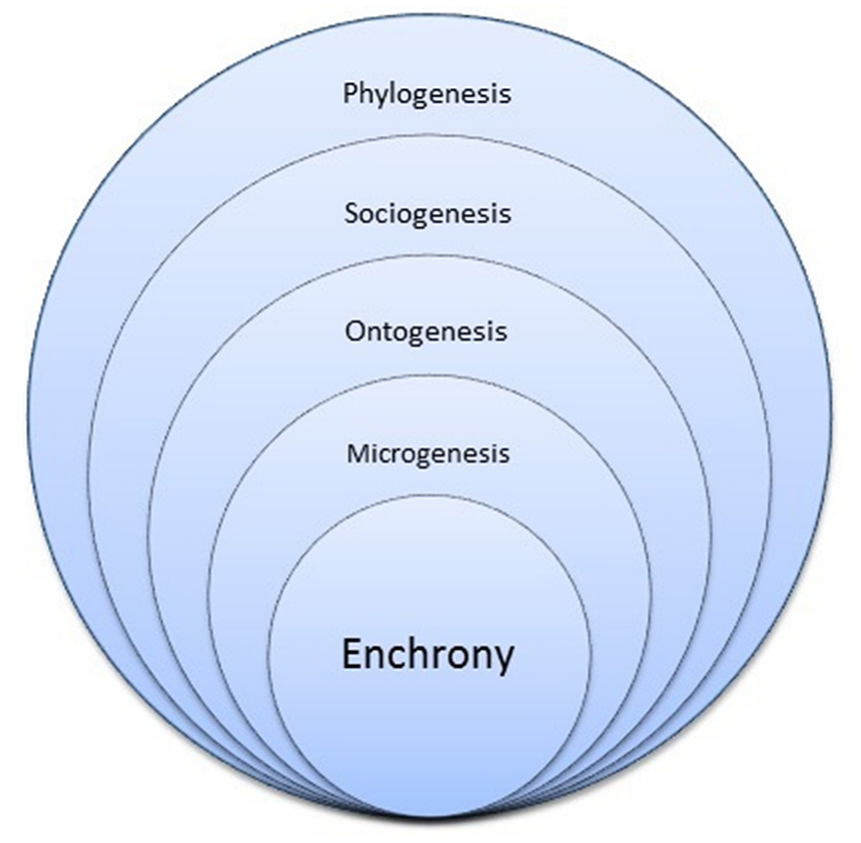
**Time scales of human evolution and development.** Figure © Chris Sinha.

We can visualize the different, but inter-articulated time scales (phylogenesis, sociogenesis, ontogenesis, microgenesis, enchrony) as being embedded one within another (**Figure 2**). Each level provides a context and platform for the levels that are subsequently embedded, but this dependency is not one of unidirectional determination. The processes that dynamically unfold in the different time scales are not independent of each other. Ontogenesis, for example, evolves phylogenetically and, through epigenesis and Baldwin effects (Sinha, 1988, Ch. 4), phylogenetic evolution itself is mediated by ontogenetic development. Microgenesis implicates and is supported by enchrony. My argument, then, is that human infancy and childhood is a developmental (and developing) niche that evolved in our species, through a mechanism of augmented epigenesis throughout an extended human life course, as an adaptation to the enchronic properties of human action and interaction. This adaptation in turn maximized the human microgenetic learning potential underlying the cultural transmission of both language and skilled praxic action.

Augmented epigenesis in the time-extended human ontogenetic niche furnished, I suggest, a phenogenotypic mechanism suitable for stabilizing and expanding the diversification and innovation in material and symbolic cultures that first appears in the archeological record in Southern Africa in the Middle Stone Age, developing between 100,000 and 75,000 years ago (Henshilwood and Dubreuil, 2011; Henshilwood et al., 2011). This diversification and innovation is taken by many archeologists to be the hallmark of “behavioral modernity.” Criterial for behavioral modernity is the existence of a symbolically mediated culture, in which “individuals understand that artifacts are imbued with meaning and that these meanings are construed and depend on collectively shared beliefs,” which in turn “explains how human norms and conventions differ from the ritualized behaviors found in non-human primates” (Henshilwood and Dubreuil, 2011, pp. 368–369).

When did such normative meaning construal emerge in the evolutionary history of the species? There is evidence of the engraving by late *Homo erectus* of quasi-geometric designs on shells, around half a million years ago (Joordens et al., 2014). These findings compel the conclusion that the time depth of the co-evolution of semiosphere and technosphere is far greater than that of our species. Do they also compel the conclusion that evolutionarily modern, complex language might be equally old? Just such a hypothesis has been advanced by Dediu and Levinson (2013), who place the dawn of evolutionarily modern language at around half a million years ago, with the common ancestor of modern humans and Neanderthals. They suggest, furthermore, that present-day languages have traces of the admixture of Neanderthal languages, just as the genomes of some present-day human populations exhibit traces of interbreeding with Neanderthals.

There is indeed evidence that Neanderthals manifested some of the practices clustered together as behavioral modernity, including personal ornamentation and funerary practices (d’Errico et al., 2012). It is highly likely that their communicative capacities included multimodal proto-language. However, there is at present no evidence that Neanderthals (or any common ancestor of Neanderthals and modern humans) maintained material-symbolic “techno-complexes,” of the kind inferable from archeological remains of modern human populations in Southern Africa, over extended periods. It is also noteworthy that innovations introduced by Southern African modern humans in the Middle Stone Age, some 80,000 years ago, disappear from the archeological record at about 60,000 years ago, only to be superseded by new innovations appearing some 15–20,000 years later. Some of these later innovations (from about 44,000 years ago) manifest apparent continuity with historic hunter-gatherer societies (d’Errico et al., 2012). It seems, then, that the human cultural “ratchet effect” (Tomasello, 1999), by means of which cultural innovations are preserved, transmitted and serve as the foundation for subsequent innovation, may not have been fully in place until quite late in the evolution of our species.

If we accept with (Smith et al., 2007a,b) that the modern human life course was not shared by Neanderthals, it is a plausible inference that the augmented epigenetic developmental plasticity of our species was also not shared in the same degree by any other (extinct) hominin species, but evolved either as part of, or subsequent to, modern human speciation. What specific advantage, then, might species-specific augmented epigenesis have conferred, and with what consequences? Perhaps the decisive selective advantage conferred by augmented epigenesis had not only to do with the faithful copying and transmission of specific material and symbolic practices, important as this may be; but also with the role of learning and teaching in generating and tracking cycles of expansion and stabilization of the symbolic space of social and cultural structure, brought into being by the evolution of the all-pervasive, linguistically grounded semiosphere. We can think here, perhaps, of the development of socio-cognitive logics such as kinship systems and elaborate cosmologies. All of these involve a level of linguistic complexity, involving flexible cognitive construal (Langacker, 1987) and narrative (Bruner, 1990; Talmy, 2000), only to be found in fully evolutionarily modern, speech-based and highly grammaticalized languages.

**FIGURE 3:**
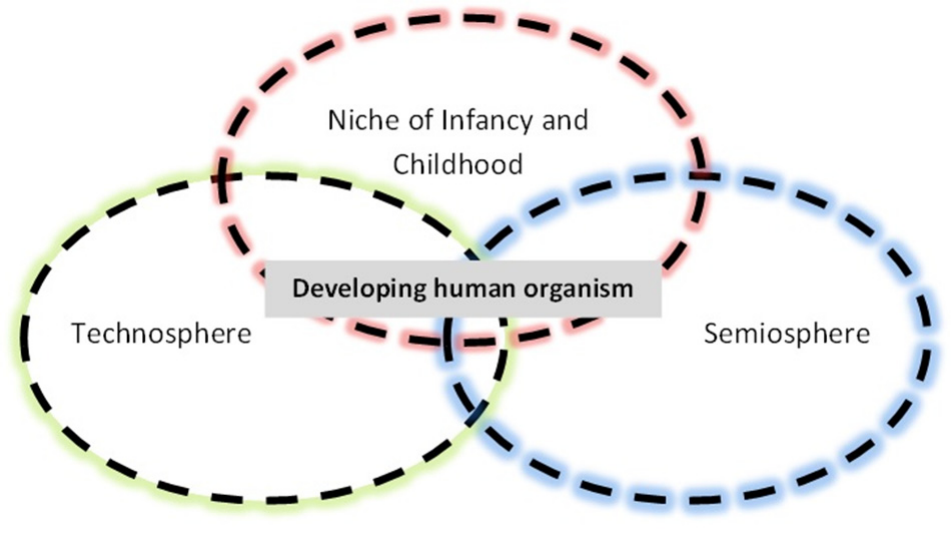
**Niche-organism-niche co-evolution of the human biocultural complex.** Figure © Chris Sinha.

My proposal, in summary, is that not only did the emergence of the *Homo sapiens* species deliver a uniquely “language-ready brain” (Arbib, 2012), but this brain was itself the product of a “language-ready niche of development,” whose phenogenotypic co-evolution with the human organism led to the marrying of augmented epigenesis with the linguistically mediated teaching and learning of social symbolic knowledge at some point in the last 160,000–80,000 years. The evolutionary intertwining of semiosphere and technosphere extends back in evolutionary time at least half a million years, perhaps longer, and the modern human mind is a product of niche-organism-niche interactions, in which niche-niche synergies were *mediated* by the organism and organismic behavior (**Figure 3**).

I have emphasized in this section the key role played by the evolution of the capacity for enchronic communicative coordination in the co-evolution of semiosphere and technosphere, but this is not intended as a claim for its exclusive importance in the evolving suite of human socio-semiotic abilities. For example, internalized iconic narrative representations of sequences of contiguous episodic memories is likely to have been a key prerequisite both for the emergence of language (Stutz, 2014), long predating complex and perspectivized narratives; and for the learning of complex sequences of constructive praxic action. The more general point is that the co-evolutionary dynamic linking semiosphere and technosphere was fundamentally transformed by the emergence of evolutionarily modern languages as the fundamental ground of symbolically mediated social institutions. Grammaticalization based upon perspectivization, alternate construal, markers of intersubjectivity/alterity (Danziger and Rumsey, 2013) and many other socio-cognitive dimensions (Talmy, 2015) made possible the elaboration of kinship relationships, mythic narratives, cosmologies and the ritual enactment of socially differentiated belief and knowledge systems. It is as a consequence of this final “leap to language” (which I hypothesize to have occurred *after* modern human speciation) that the ground was laid for the emergence of symbolic cognitive technologies that have fundamentally re-shaped (and continue to re-shape) the human biocultural complex.

## Beyond Linguistic Relativity: Time In Mind, Culture, And Society

**FIGURE 4:**
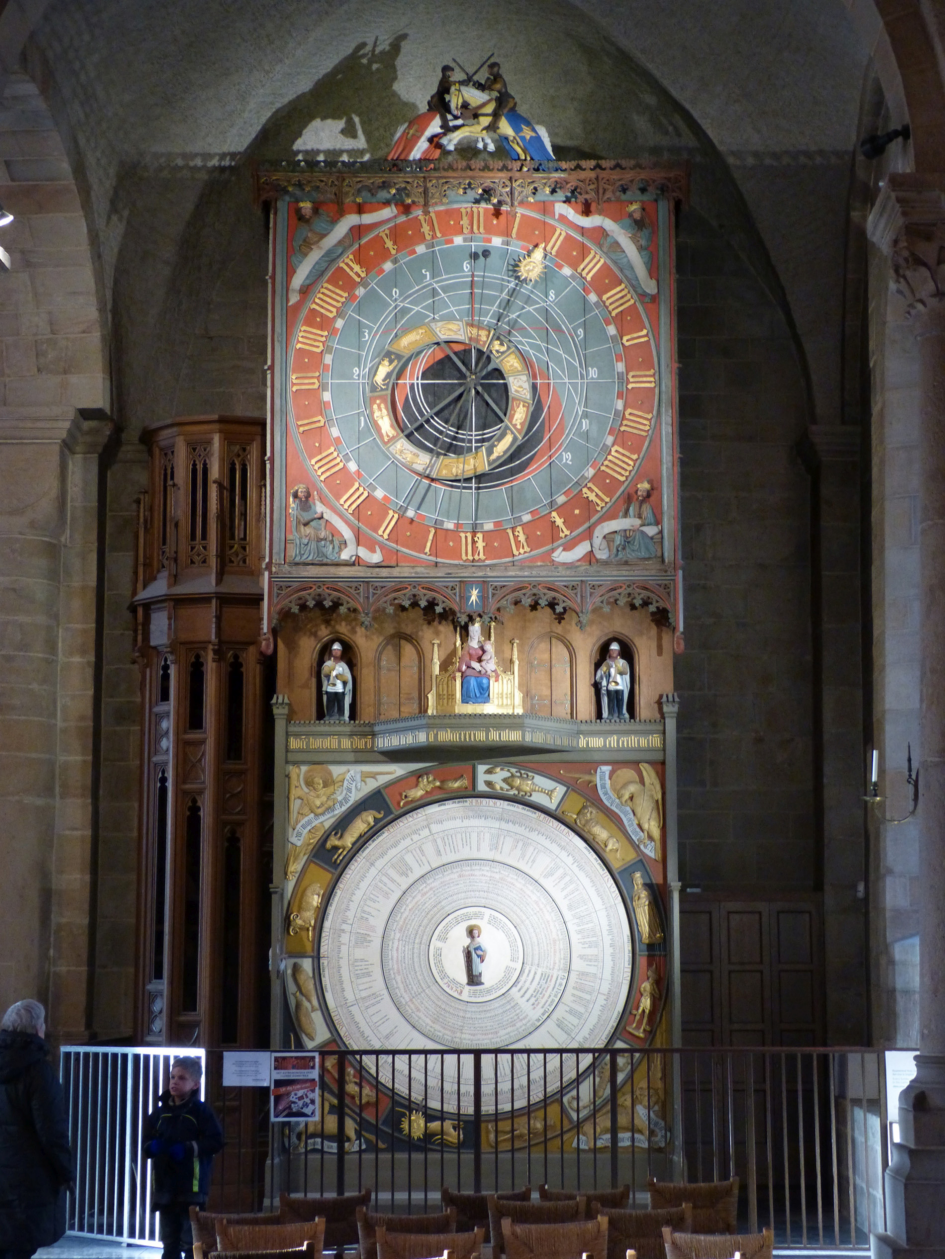
**A restored medieval clock in Lund Cathedral, Sweden.** Image © Chris Sinha.

A striking exemplar of a historically important symbolic cognitive artifact type is the medieval clock shown in **Figure 4**. Such clocks are scattered throughout North-West and Central Europe. Early church and cathedral clocks lacked faces, and sounded the hours by the ringing of bells (Whitrow, 1988), but later ones incorporated clock faces schematically representing cyclic time intervals—in the case illustrated, not only the hours of the day, but also months and years. The circular form of the clock face iconically represents the cyclic schema which organizes the numerically (ordinally) based time intervals. Although clock hours and calendar intervals are a much older invention than the mechanical clock itself, dating to the Babylonian civilization, these time intervals were dependent upon number notation, as well as upon the astronomical observations measured and notated. Number notations themselves are derived from linguistic number systems whose origins are to be found in counting practices. So there is a fundamental ambiguity about the notion of “clock time”: is it that which is *measured by* the instrument, or is it the calibrations that are produced by the mechanical or other operations of the measuring artifact? In fact, these two aspects are, from a sociogenetic point of view, two sides of the same coin: only by constructing symbolically based, time-calibrating systems do societies arrive at a conceptualization of time as a “dimension” (or “timeline”) that can be measured; and this conceptualization is in turn embodied in *material anchors* (Hutchins, 2005) that both represent the conceptualization, and permit or improve the measuring practice.

The entrenching of “calendar time” (important for the computation of saints’ days), and later “clock time,” had profound consequences for medieval and early modern European societies. It enabled the accurate determination and registration of both religious festivals and secular time. The consequences of the invention and cultural evolution of the calendar and the clock have been no less transformative for the mind than for the social world. Concomitantly with the historical invention of more accurate ways of measuring metric time intervals, a new concept of time of time evolved: abstract “Time as Such.” By this, I mean that notion of time that metaphorically situates or encompasses the events that occur “in time,” and their time of occurrence, analogously to the way that space situates or encompasses objects and their locations. “Time as Such” is the pre-theoretical equivalent of what was referred to by Newton (2009/1686) as “Absolute Time.” Newton’s (2009/1686) postulation of the metaphysical reality of Absolute Time was famously challenged by Einstein’s theory of relativity (Einstein, 1920), but “Time as Such” is deeply rooted in everyday language practices. It can be thought of as *reified time*, conceptualized as an abstract but quantifiable substance with a quasi-spatial extension. This substance either metaphorically “flows” (moving along a timeline in relation to an observer), or is a medium, landscape or timeline within or along which the observer moves. These two metaphoric conceptualizations are generally referred to as “Moving Time” and “Moving Ego” (Clark, 1973), and they are commonly manifested in many languages in such constructions as, respectively, “Graduation Day is approaching” and “she is coming up to graduation.”

The supposed absence of the “Newtonian” notion of “Time as Such” in some cultures was key to Whorf’s (1950) celebrated “principle of linguistic relativity.” Speakers of the Amerindian language Hopi, he maintained, have “no general notion or intuition of time as a smooth flowing continuum in which everything in the universe proceeds at an equal rate, out of a future, through a present, into a past; or, in which, to reverse the picture, the observer is being carried in the stream of duration continuously away from a past and into a future” (Whorf, 1950, p. 27). Whorf (1950) did not publish extensive linguistic data that could underpin this claim; those which he did publish were open to the objection of being idiosyncratically interpreted, and his analysis of the Hopi language has been challenged in subsequent, more rigorously documented scholarship (Malotki, 1983)^5^. However, my colleagues and I conducted a study of an Amazonian language and culture, Amondawa, in which [as Whorf (1950) maintained was the case for Hopi] there is no translation equivalent for the word “time,” no evidence of a cultural concept of “Time as Such” and no use of metaphoric Moving Ego or Moving Time constructions (although bilingual Amondawa speakers understand such constructions in Portuguese; Sinha et al., 2011). Crucially, the Amondawa language has only four numbers and does not employ a calendric system. All Amondawa time interval concepts are event-based (da Silva Sinha et al., 2012), that is they are non-metric and are derived from the duration and succession of natural events, and the conventional timing and duration of habitual social activities^6^.

Our research on cultural and linguistic concepts of time in Amondawa provides a partial vindication of Whorf’s (1950) insistence that “Newtonian time” (or what I have termed “Time as Such”) is not a transcultural universal. Our findings, however, are not consistent with a construal of the “linguistic relativity” of thought as involving a two-term relationship between linguistic structure and individual cognitive process. Rather, and in line with the arguments advanced by Palmer (1996), we would view languages as reflecting and entrenching culturally specific patterns of thought that also find expression in other semiotic vehicles. In particular, Sinha et al. (2011, p. 165) advance the Mediated Mapping Hypothesis, according to which the “elaboration of (space-time metaphoric) mapping is mediated by number concepts and number notation systems, the deployment of which in symbolic cognitive artifacts such as calendar systems transforms the conceptual representation of time from event-based to (metric) time interval systems; yielding the culturally constructed concept of ‘Time as Such.’

This re-interpretation of the issues surrounding linguistic relativity is, I suggest, in tune with the Zeitgeist. The “cognitive turn” in language sciences of the past 25 years is now being followed by a “cultural turn” (Everett, 2012; Bernárdez, 2013), highlighting the dynamic and mutual inter-relations between language, cognition and culture, and their co-variation. The sociocultural contextualization of language and cognitive diversity in our and others’ recent work, together with a general theoretical perspective of extended material-symbolic cultural embodiment, points the way to a new, post-Whorfian perspective on the inter-relations between language, cognition and culture, and their co-variation (Sinha and Bernárdez, 2015). A core theoretical construct of this post-Whorfian perspective is what Birth (2012, p. 19) dubs ‘artifactual relativity’: “cognitive artifacts have far greater potential for channeling thought than language … clocks and calendars being two important examples. In effect, whereas the strength of the linguistic relativity hypothesis is hotly contested, a parallel hypothesis about artifactual relativity has received little attention”^7^.

The phenomena studied through the lens of artifactually mediated relativity, or variation, may be prototypically instantiated by symbolic cognitive artifacts, but they do not stop there, and may also profitably be the subject of future investigation at a more macro-social scale. Methodologically, this means that we need to step back from our preoccupation with the “local,” the here-and-now of experimental situations, in our explorations of the language of space and time. Theories of language variation now recognize the prevalence of persistent, lexically widely distributed language-specific patterns of semantic motivation, or “semplates” (Levinson and Burenhult, 2009). This approach can be taken a further step forward, by extending the linguistic analysis of recurrent, culturally motivated pattern to the constructional and metaphoric levels, and seeking motivations that unify different levels of meaning within diverse multi-level, material-symbolic socio-cognitive niches.

The sociocultural structuring of space and time is achieved by practices involving the construction and use of artifacts and artifact systems that blend the material and the symbolic at different scales. These include the kind of symbolic cognitive artifacts that have been in focus in this article, such as compasses, clocks, calendars, and other time interval systems based on language. Material symbolic mediators also include, however, the built environment (such as architecture and village and city layout); and the humanly shaped landscape (such as geomorphic earthworks). The meanings of these material-symbolic systems range from the expression of social differentiation (gender, rank, clan, moiety etc.) in spatial and temporal dimensions; through architectural renderings of cosmological and religious beliefs; to the spatio-temporal ordering of normatively organized activities by means of time-reckoning artifacts.

These considerations challenge the assumptions that have guided many investigations of linguistic space-time mapping, which have been overly (if unconsciously) constricted by investigators’ own cultural experience and cultural concepts of time. We need to recognize that the time that we inhabit is an artifact, a fiction in a way, which is itself the product of the artifacts that our ancestors have invented. Time, we might say, is a cognitive meta-niche, a necessary regulative order for the reproduction of the multiplicity of other cognitive-cultural-material niches that support our activities, practices, communications and reflections. But it is simultaneously a cognitive construct, assembled through the spatialization and reification of temporal experience. When employed to regulate social and economic life, clock and calendar impose a fictive and conventional structure on mundane, terrestrial event time, “freezing” temporal passage into regimes of activity-mapping and time-planning.

The reifying fiction of “Time as Such” is entrenched in conventional metaphors and idiomatic usage, in which “time is money,” “time is scarce,” people are time-poor, and time endlessly presses up against us. The symbolic cognitive artifacts of clock and calendar have changed our minds along with the niches our minds inhabit, and there is no going back in time. These artifacts, and the language practices that they support and constrain, are fundamental to the regulation and reproduction of every social institution in which we participate. The first, naïve response of many people to hearing there still exist societies in which these artifacts are non-existent, and in which all time intervals and temporal landmarks are event-based, is to ask: does that mean they have no idea of time? How can they organize their lives? And yet, a moment’s reflection will tell us that the event-based *habitus* of the Amondawa is representative of the conceptual matrix framing temporal experience that has been characteristic of the majority of human societies. Human beings have lived in small-scale, face-to-face, technologically simple societies for most of the history and prehistory of our species. It is our fast-tracked, globalized, 24/7 turbo-capitalist society that is the exception. Artifactual “Time as Such” has colonized the niche, and the niche in turn has colonized our minds.

## Concluding Reflection: Agency Vs. Artificiality—A False Dichotomy?

The linguistically constituted human semiosphere is a species-specific biocultural complex, grounded in the elaboration of the semiotic function (Piaget, 1945). This function, in turn, is constituted by the interplay and evolutionary-developmental interlacing of its two constituent semiotic relations, “counting as” and “standing for.” These two semiotic relations are, I suggest, evolutionary derivatives of *ritualization* and the evolution of *symbols from signals* (Sinha, 2004); in which the conventionalization of intersubjective participation in niche-regulated activities played a central role (Sinha and Rodríguez, 2008). The evolution of the human semiosphere, in which language as a biocultural niche is developmentally and processually interdependent with the technosphere of material artifactual supports for human social interaction and social practice (Sinha, 2005), is what accounts for the discontinuity dividing human from non-human cognition and culture, and the evolutionary emergence of human social institutions. This discontinuity has been amplified by the consolidation, through language, of human culture as a fundamentally symbolic biocultural complex. A critical role in this consolidation was played by the co-evolution of the biocultural niche of infancy and childhood with the biocultural niche of language.

If (as I have argued) symbolic cognitive artifacts have the effect of changing both world and mind, is it enough to think of them as mere “tools” for the realization of human deliberative intention, or are they *themselves* agents? This question would be effectively precluded by some definitions of agency, such as that to be found in Barandiaran et al. (2009, p. 3), who state that “as opposed to other systems, agents appear as unified in themselves and do not depend on their being useful for an external entity or accorded on (*sic*) their identity by a community of other agents.” A problem with this generalized approach, that seeks to define agency in terms that span biology and Artificial Intelligence, is that, in emphasizing the distinction between agent and environment, and contrasting agents with artifacts, it fails to engage with the complex network of mediation of distinctively human, social agency by artifactual means. It is precisely the importance of this network for both cognitive and social theory that Latour (1996) highlights by introducing the concept of “interobjectivity.” In a complementary fashion to the way that the term *intersubjectivity* denotes the sharing of the subjective aspect of agency with other subjects (Zlatev et al., 2008), *interobjectivity* denotes the way in which agents “share the social with things … Objects do *do* things … one can never reduce or dissolve an actor into a field of forces or into a structure. One can only share in the action, distribute it with other actants.” (Latour, 1996, pp. 235–237). In other words, the specifically social form that human agency takes is based not only upon social interactivity with other agents (De Jaegher and Froese, 2009)—which Latour (1996) argues cannot alone provide a solution to the “structure vs. agency” problem in social theory—but also upon its distribution amongst human and artifactual actors: “(for) humans it is almost impossible to find an interaction that does not make some appeal to technics” (Latour, 1996, p. 238). It is precisely this notion of “sharing sociality with things” (*ibid*: 237) that I stress by introducing the term “technosphere”

Many discussions of distributed and extended cognition focus on the effects of artifacts on cultural evolution in terms of the externalization of information storage, and the enhanced accuracy of transmission of knowledge and social memory (Donald, 1991). I have argued that this, while important, is not the whole story. Symbolic cognitive artifacts are not just repositories, they are also *agents of change*, constituting new domains (such as “Time as Such”), and potentiating new practices. We can acknowledge that the agency of artifacts is (at least until now) ultimately dependent on human agency, without which artifactual agency would neither exist nor have effect; but it would be wrong to think of artifactual agency as merely derivative, as being like a kind of glorified transmission-belt for human agentive intention. Human agency, in many cases, is co-agency, not only with other human beings but with at least some kinds of artifacts. Co-agentive artifacts play an ever-expanding role in the human biocultural complex, and assume increasingly autonomous modes of agency. This poses a real challenge both to our understanding of the nature of knowledge and to our understanding of the nature of ethical and social responsibility in science. More than that, the challenge is potentially an existential one to the future of our species in its self-made ecology.

## Conflict of Interest Statement

The authors declare that the research was conducted in the absence of any commercial or financial relationships that could be construed as a potential conflict of interest.

## Footnotes

1 “Symbol” and “symbolic” are notoriously polysemous and contested concepts. In accordance with Bühler’s (1990/1934) classification, symbolicity is here understood in terms of the semiotic, pragmatic and intersubjective logic of communicative representation, not on the typology in the Peircian sense (Peirce, 1931/1958) of the relationship between sign and object (see also Sinha, 1988, 2004).

2 Iconic signs, such as pictures, may also have a representational function, but are not necessarily cognitive artifacts, since their function may be entirely aesthetic. Maps, which are cognitive artifacts, are often regarded as iconic signs, but they depend not only on the iconic representation of the territory, but also upon the linguistic naming of places within it; and their use is always within the intersubjective field of a universe of discourse. In Bühlerian terms, they are (like Chinese ideographs) *iconically motivated symbolic forms*.

3 The “ratchet effect” consists in the cumulative intergenerational transmission of cultural innovation, and appears to be unique to the human species. Its effective operation may also depend upon the demographic factor of population size (Dediu and Levinson, 2013).

4 The formalisms and argumentative framework in the remainder of this section were first presented in Sinha (2009).

5 Malotki’s analysis has also been challenged on the grounds that it does not adequately address the (admittedly idiosyncratically and at times opaquely expressed) theoretical propositions advanced by Whorf (Hinton, 1988).

6 Amondawa is not the only language in which the absence of a cultural concept of “Time as Such,” and of its anchoring in a calendar or timeline, is correlated with the absence or lack of productivity of space-time metaphoric mapping (Le Guen and Pool Balam, 2012; Levinson and Majid, 2013).

7 Birth arrived at this formulation, on the basis of his research in the anthropology of time, entirely independently of the similar arguments about artefactual mediation proposed by Sinha et al. (2011).
